# Lifetime management of primary mitral regurgitation through integrated surgical and transcatheter reinterventions

**DOI:** 10.1038/s44325-026-00129-2

**Published:** 2026-05-25

**Authors:** Minoru Tabata

**Affiliations:** Department of Cardiovascular Surgery, Juntendo University, Tokyo, Japan

**Keywords:** Cardiology, Diseases, Medical research

## Abstract

Lifetime management of primary mitral regurgitation requires integrating surgical and transcatheter strategies. This review evaluates initial interventions, emphasizing early timing and repair durability, and outlines subsequent reintervention pathways. Synthesizing contemporary evidence, it highlights how a multidisciplinary Heart Team must design the index procedure to preserve future therapeutic options and ensure structured longitudinal care across the patient lifespan.

## Introduction

The “lifetime management” paradigm has transformed treatment planning for aortic stenosis (AS), integrating age, anatomy, durability, and reintervention pathways (e.g., surgical aortic valve replacement → valve-in-valve transcatheter aortic valve replacement [TAVR]; TAVR → redo TAVR or explant). A similarly thoughtful approach is now needed for primary mitral regurgitation (PMR). For many patients with PMR, the ideal scenario is a single, durable surgical repair that achieves lifelong valve competence. However, not all patients are appropriate candidates for surgical repair due to operative risk or valve morphology, and not all repairs remain competent indefinitely; some will ultimately require reintervention. As a result, lifetime management in PMR must incorporate both the pursuit of a durable index operation and the foresight to preserve future options should reintervention become necessary.

The 2025 ESC/EACTS Valvular Heart Disease Guideline includes several updates with important implications for lifetime management of MR: (1) refined indications for early surgery in PMR; (2) practical criteria for M‑TEER patient selection; and (3) expanded recommendations for prosthetic failure including mitral valve‑in‑ring (ViR) and mitral valve‑in‑valve (ViV) at intermediate/high surgical risk^1^.

This review synthesizes contemporary evidence to present a practical approach to lifetime management of PMR. Key topics include first-line therapy, reintervention options (redo repair, redo MVR, TEER, transventricular beating-heart chordal implantation, ViR, and ViV), and initial treatment strategies designed with future interventions in mind.

## Diagnostic framework for primary MR

PMR comprises a spectrum of primary valvular abnormalities ranging from fibroelastic deficiency (FED) to Barlow’s disease, with many patients presenting intermediate (“forme fruste”) features. FED typically involves isolated prolapse due to chordal rupture with thin, translucent leaflet tissue and normal annular size, whereas Barlow’s valves show diffuse myxomatous thickening, multi-segment prolapse, enlarged annuli, and redundant leaflets. Forme fruste morphology falls between these extremes^2^. Although these categories help conceptualize PMR, they represent a continuum rather than discrete entities and require individualized repair strategies.

Echocardiography (2D and 3D, transthoracic and transesophageal) remains the foundation of morphologic and mechanistic assessment. Key elements include the identification of prolapsing or flail segments, leaflet redundancy, coaptation abnormalities, the severity and origin of the regurgitant jet, annular dilation, annular displacement or disjunction, annular or leaflet calcification, left ventricular morphology, and leaflet tethering. Cardiac MRI can assist with regurgitant-volume quantification, and CT is used selectively for preoperative planning or redo cases, and planning of some transcatheter procedures.

Finally, mixed pathology is common, especially in elderly patients who may exhibit PMR alongside atrial enlargement, annular dilatation, or LV remodeling consistent with atrial or ventricular secondary MR mechanisms. Although this review focuses on PMR, such mixed presentations require a heart-team approach that integrates treatment strategies for PMR, atrial secondary MR, and ventricular secondary MR when planning lifetime management.

## Timing of intervention: evolving concepts in asymptomatic PMR

Early intervention in asymptomatic PMR has long been supported by multiple lines of evidence^3–7^. More recently, accumulating data have further refined the criteria for intervention, highlighting that timely repair optimizes long-term outcomes and that waiting for symptoms or left-ventricular dysfunction may reduce the likelihood of achieving a durable correction^8,9^.

The 2025 ESC/EACTS Valvular Heart Disease Guideline introduces several practice-defining updates relevant to this population. Surgery is recommended (Class I) for asymptomatic severe PMR when conventional thresholds of left-ventricular dysfunction are met (LVESD ≥ 40 mm, LVESDi ≥20 mm/m², or LVEF ≤ 60%). The guideline also introduces a *new* Class I indication: surgery is recommended in asymptomatic patients when three or more of the following are present—atrial fibrillation, pulmonary hypertension, marked left-atrial enlargement, or concomitant moderate or greater secondary tricuspid regurgitation^1^. These criteria reflect clinical markers of disease progression and downstream consequences of chronic MR rather than leaflet pathology itself.

Beyond these triggers, early surgery is considered appropriate (Class IIa) in low-risk asymptomatic patients when the likelihood of achieving a durable repair is very high—a recommendation that has been retained from prior guidelines^1,10–12^. In patients with favorable anatomy, especially when treated at experienced centers where repair success exceeds 95% (although quantifying this likelihood preoperatively is not straightforward), delaying intervention may allow progressive LV remodeling, atrial fibrillation, or pulmonary hypertension to develop, each of which has been associated with higher rates of recurrent MR after repair.

## First intervention: surgical repair principles for long-term durability

Surgical repair is the first-line treatment in PMR, and achieving a durable repair is the single most important determinant of successful lifetime management. This section reviews the principles and techniques that underpin durable repair, integrating contemporary evidence and expert consensus.

Annuloplasty is essential for the long-term durability of degenerative mitral repair. A prosthetic ring or band, secured from trigone to trigone, restores normal annular size and shape, deepens leaflet coaptation, and prevents future dilatation of the unsupported posterior annulus. Omission of annuloplasty greatly increases recurrence risk^2,13,14^. In practice, the ring type (complete vs. partial band) and rigidity (rigid, semi-rigid, semi-flexible, flexible) have less impact on durability than proper sizing and implantation technique^15–17^. All annuloplasty rings must be secured to the fibrous portion of the annulus, including both fibrous trigones. Complete rings provide circumferential support against dilation, whereas posterior bands preserve annular motion. Rigid designs offer stronger remodeling, while flexible designs maintain physiologic dynamics. Accurate sizing is the key. It is common to size the annuloplasty ring to the anterior leaflet’s area or height, or to the inter-commissural distance, rather than aggressively undersizing in PMR. The selected ring size may shift depending on posterior-leaflet size or left-ventricular dimensions. A ring that is too small increases the risk of mitral stenosis (MS) or systolic anterior motion (SAM), whereas an excessively large ring may compromise leaflet coaptation.

Leaflet management is tailored to lesion anatomy. Both leaflet resection and artificial chordal replacement can be durable when executed well. Recent series suggest that resection may yield lower late reintervention than chordal replacement in PMR^18^, yet chordal replacement can deepen the coaptation point^19^, and greater coaptation length itself has been associated with lower rates of recurrent MR^20^. In practice, careful chordal length setting to anticipate LV remodeling may be important, and combining limited resection with artificial chords over the most degenerated segments can capture the advantages of each approach while maintaining a broad, ventricularly positioned coaptation.

Avoiding iatrogenic MS and SAM is essential for durable PMR repair. Functional MS results from excessive leaflet resection, extensive edge-to-edge stitching or multiple indentation closures, or from an undersized annuloplasty and the use of a rigid complete ring that restricts diastolic opening^21–23^. SAM, in contrast, reflects systolic leaflet drag toward the LVOT when the coaptation point is too anterior or leaflet heights are disproportionate. It is prevented by achieving an appropriate anterior-to-posterior leaflet height ratio, maintaining a deep and posteriorly positioned coaptation line, and avoiding excessive downsizing^24^. If SAM is detected intraoperatively, it is corrected with posterior-leaflet height reduction, modest ring upsizing, chordal length adjustment, or a focal edge-to-edge stitch^25,26^. Through precise ring sizing, thoughtful leaflet-height management, and rigorous intraoperative evaluation of gradients and LVOT flow, surgeons can minimize MS and SAM, two preventable complications that directly influence the durability and lifetime success of PMR repair.

Before closure, confirm on TEE that MR is no more than mild, the regurgitant jet is not eccentric, coaptation is broad and symmetric, mean gradient remains within safe limits, there is no LVOT obstruction, and ring seating with trigonal anchoring is stable^2^. Early failure typically reflects under-correction or technical issues (residual prolapse, inadequate coaptation, ring dehiscence); late failure most often reflects progression in untreated segments, artificial chord elongation, or iatrogenic stenosis. Even after an excellent repair, periodic echocardiographic follow-up is essential to detect recurrent MR before adverse remodeling; a well-balanced index reconstruction both lowers the probability of reintervention and preserves options if reintervention is needed.

Across contemporary series with 10 years or more of follow-up, surgical repair for degenerative MR shows excellent echocardiographic durability: in the 1234-patient cohort, the probability of recurrent moderate or severe MR was 12.5% and reoperation-free survival was 60.4%^27^ Earlier lesion-specific analyses reported posterior repairs achieving 80–90% freedom from moderate or greater MR at 10–12 years, compared with 65–75% for anterior and bileaflet prolapse^28,29^. However, a recent propensity-matched study of 1025 degenerative repairs found no significant differences between anterior (including bileaflet) and isolated posterior repairs in residual MR or 15-year reoperation rates (7.5% vs 4.9%), indicating broadly comparable long-term clinical durability across leaflet types in experienced centers^30^.

Beyond the specific location of the prolapse, several clinical, morphological, and procedural factors strongly dictate the risk of late MR recurrence after an initially successful repair. Established patient-related and structural predictors of recurrent MR include older age, advanced myxomatous degeneration (lesion complexity), preoperative atrial fibrillation, left ventricular (LV) enlargement, reduced ejection fraction, and pulmonary hypertension^8,27,31^. Furthermore, regarding subtle LV dysfunction, reduced myocardial strain specifically in the papillary muscle region has been reported to correlate with repair failure^9^. From a technical standpoint, the omission of an annuloplasty ring and the acceptance of even mild residual MR at the conclusion of the procedure substantially increase the hazard of late recurrence^27,32,33^. Crucially, many of these preoperative risk factors reflect a more advanced stage of disease that can be effectively mitigated by operating earlier in the disease course. Therefore, the importance of early intervention is reinforced not only by long-term survival benefits but also by the significantly higher probability of maintaining lifelong repair durability. Intraoperatively, these recurrence predictors validate the necessity of employing a prosthetic annuloplasty ring and maintaining a strict, zero-tolerance approach toward residual MR.

## Other options as index intervention

Although surgical repair remains the first-line treatment in PMR, several alternative strategies may serve as the initial intervention in highly selected circumstances.

### Mitral valve replacement (MVR)

MVR is reserved for valves that are not repairable, such as those with extensive leaflet calcification or restriction, multi-segment involvement with limited leaflet tissue or extensive infective endocarditis. MVR provides predictable elimination of MR and acceptable short-term operative outcomes, although perioperative mortality and morbidity remain higher than with repair. Long-term outcomes are also less favorable: mechanical valves offer structural durability but require lifelong anticoagulation, while bioprostheses avoid anticoagulation but undergo structural degeneration within 10–20 years^34,35^, necessitating lifetime planning for redo MVR or ViV procedures.

In young patients (typically <60–65 years), prosthesis selection represents a particularly complex dilemma within the lifetime management framework. While clinical guidelines traditionally favor mechanical valves in this age group to avoid multiple reoperations^1,10,11^, there is a growing trend of young patients opting for bioprostheses to avoid lifelong anticoagulation, largely driven by the expectation of future ViV interventions. However, it must be strongly emphasized that structural valve deterioration is significantly accelerated in younger patients. Compared to mechanical valves, the use of bioprostheses in this demographic is associated with both a higher cumulative risk of reoperation and significantly higher long-term mortality^36^. Consequently, implanting a bioprosthesis in a young patient almost certainly guarantees the need for multiple reinterventions over their lifespan. Given the anatomical and hemodynamic limitations of performing multiple ViV procedures (as discussed in detail in the subsequent sections), these patients may ultimately require a high-risk redo surgical MVR later in life. Therefore, prosthesis selection in young patients must not rely on overly optimistic views of transcatheter options but requires rigorous, Heart Team-guided shared decision-making that outlines a realistic, multi-stage lifetime plan.

### Transcatheter edge-to-edge repair (TEER)

TEER is generally reserved for symptomatic severe PMR patients with prohibitive or high surgical risk and favorable grasping anatomy. In a contemporary U.S. degenerative MR registry (19,088 patients), 1-year MR success was 88.9%, mortality 15.4%, and reintervention 3.4%, with the best outcomes when residual MR ≤ mild and mean gradient ≤5 mm Hg (1-year mortality 11.4%)^37^. Randomized data confirm that long-term durability remains inferior to surgical repair: in EVEREST II, the 5-year effectiveness composite was 44% after TEER vs 64% after surgery, driven by higher late recurrence and reoperation in the TEER arm^38^. With contemporary devices, CLASP IID showed sustained mid-term MR reduction in high-risk PMR (MR ≤ 2+ at 2 years: PASCAL 95% vs MitraClip 91.5%) with stable gradients^39^.

Durable TEER in PMR requires optimal anatomy, precise imaging guidance, and favorable hemodynamics. Ideal features include a central A2–P2 jet, posterior-leaflet length ≥10 mm, flail gap ≤10 mm, flail width ≤15 mm, and MVA ≥ 4.0 cm². Procedure success depends on coaxial 3D-TEE-guided positioning, independent leaflet grasping, and avoiding excessive clip stacking. Completion targets are MR ≤ 1+ and mean gradient ≤5 mm Hg, and creation of a broad, posteriorly positioned coaptation zone, as adequate coaptation reserve predicts sustained MR reduction and improved survival^40,41^.

### Transcatheter mitral valve replacement (TMVR)

For patients with severe PMR at prohibitive surgical risk and anatomies unsuitable for TEER, TMVR is emerging as a potential transcatheter alternative. While recent early feasibility trials of transfemoral systems have demonstrated excellent early safety, such as 0% 30-day mortality in a highly selected cohort of 33 patients^42^, real-world outcomes reveal a more complex reality. In a global registry of 195 patients treated with a transapical device (encompassing mixed MR etiologies), despite a 95% technical success rate, 30-day and 1-year mortality rates were 9.3% and 28.6%, respectively^43^. This discrepancy likely reflects not only the difference in access routes but also the inclusion of approximately 30% of cases performed outside the anatomical instructions for use (IFU), underscoring the paramount importance of strict patient selection. Given the critical risks of left ventricular outflow tract (LVOT) obstruction and complex anchoring challenges, TMVR’s routine use as an index intervention in PMR cannot be endorsed until ongoing pivotal trials confirm its long-term safety across broader populations.

### Transventricular beating-heart chordal implantation

This repair-based option is best suited to highly selected anatomies—classically isolated posterior-leaflet prolapse with adequate leaflet tissue and minimal annular dilation. Short-term outcomes are favorable and recovery is rapid because cardiopulmonary bypass is avoided. Mid- to long-term durability, however, is consistently lower than with conventional surgical repair. In a 100-patient series with 5-year follow-up, device success was 94% at 30 days, 92% at 1 year, and 78% at 5 years; overall 5-year survival was 83%, and severe MR recurrence at 5 years was 14% in anatomically favorable valves versus 63% in unfavorable valves, with reintervention rates of 14.7% and 43.4%, respectively^44^. Propensity-matched comparison versus conventional repair showed inferior 5-year freedom from MR ≥ 2+ (57.6% vs. 84.6%) and from reoperation (78.9% vs. 92%) overall, though outcomes were comparable in isolated P2 disease, underscoring the importance of stringent selection^45^. A notable advantage is that preservation of the native annulus and leaflets maintains the feasibility of the full spectrum of subsequent mitral interventions—including redo repair, replacement, TEER, and future transcatheter therapies. Moreover, transseptal chordal implantation technologies are now in development, suggesting that this therapeutic class may expand in the future. However, until longer-term durability is established, these approaches should be reserved for highly selected patients with favorable anatomy and performed in experienced centers.

## Reintervention after surgical mitral valve repair

### Redo surgical repair or MVR

When operative risk is acceptable and a correctable mechanism is identified, redo repair is favored. Re-repair achieves high procedural success in expert centers with very low early mortality (0–2%) and better long-term survival than replacement^46–48^. If reparability is low (diffuse degeneration, calcification, short leaflets, complex multi-segment failure, extensive infection), MVR provides definitive MR elimination but with higher early mortality (2.6–15%)^48–50^, lower long-term survival^46–48^, and prosthesis trade-offs (lifelong anticoagulation for mechanical valves; 10–20-year structural degeneration for bioprostheses).

Reoperative exposure via right mini-thoracotomy, including robotic surgery, can limit adhesiolysis and reduce re-entry risk^51,52^; conversely, patients whose index operation was minimally invasive often have less mediastinal scarring, making subsequent sternotomy safer than after a prior full sternotomy.

### Transcatheter edge-to-edge repair (TEER)

For recurrent MR after prior surgical repair, TEER offers a less invasive option for high-risk patients, provided the leaflets are graspable, the mitral orifice is not stenotic, and there is no active infection. Across observational series, acute performance is favorable, yet durability remains uncertain, and robust long-term, head-to-head comparative data with surgical reintervention are not yet available. In an international multicenter cohort of 104 post-repair patients, technical success was about 90% and in-hospital mortality was 2%, but small or rigid annuloplasty rings were associated with clinically relevant transmitral gradients, highlighting the need to avoid functionally small orifices^53^. Editorial commentary cautions against expecting durability comparable to untreated native valves and stresses meticulous selection and intraprocedural goals, including confirming adequate ring size, hemodynamic gradients, and leaving no more than mild residual MR (ideally ≤1+) at completion^54^. Overall, TEER after surgical repair is a useful rescue strategy in selected high-risk patients, but lifetime planning should acknowledge its potentially limited durability and the ring-related risk of post-procedural MS.

### Transventricular beating-heart chordal implantation

In the reintervention setting, this repair‑based approach is best suited to highly selected anatomies: focal recurrent posterior‑leaflet prolapse, adequate leaflet length for chordal capture, a well‑seated, non‑stenotic annuloplasty ring, and no ring dehiscence, diffuse/bileaflet degeneration, or significant commissural disease. In a multicenter series specifically evaluating patients after prior surgical MV repair (15 post‑repair cases identified among 312 procedures), no in‑hospital deaths occurred; MR ≤ mild at discharge in 86.7%. “Patient success” (composite of technical success, ≤ mild MR, and freedom from death, HF hospitalization, significant MR or reintervention) was 92.3% at 1 year and 83.9% at 2 years; one high‑risk patient later died during a surgical reintervention for recurrent MR^55^. These data support feasibility, favorable early safety, and reasonable 1–2‑year durability in carefully selected post‑repair patients, while long‑term data remain limited.

### Transcatheter valve-in-ring (ViR)

Transcatheter valve-in-ring (ViR) replacement is considered for high-risk patients with failed surgical repair. Most ViR procedures use balloon-expandable valves via a transseptal approach. Ring type, size, rigidity, and geometry are major constraints: very small or rigid rings predispose to underexpansion of the transcatheter valve and subsequent high residual gradients; incomplete flexible rings may not provide secure anchoring and increase paravalvular leak or migration risk; and large anterior leaflets or large rings elevate the risk of LVOT obstruction, necessitating meticulous CT-based planning.

Contemporary registry data show 67–88% technical success (lower than ViV technical success), 30-day mortality 7–8%, and 1-year mortality 22–29%. MR reduction is generally reliable, with >90% achieving mild or less MR at 1 year, although mean transmitral gradients commonly remain elevated (6–8 mmHg), reflecting the restricted neo-orifice imposed by the ring^56–58^. While ViR reliably eliminates MR without repeat surgery, risks such as LVOT obstruction, elevated gradients, and device instability underscore the need for very careful patient and ring selection. Moreover, long-term outcomes remain uncertain.

## Reintervention after Bioprosthetic MVR

Bioprosthetic degeneration after MVR—whether due to leaflet calcification, tear, pannus, or mixed stenotic–regurgitant dysfunction—can be managed by redo surgical MVR or transcatheter mitral ViV replacement. Because redo MVR carries higher perioperative risk, valve-in-valve (ViV) has become the preferred strategy for high- or prohibitive-risk patients with suitable anatomy and no active infection.

### Transcatheter mitral valve-in-valve (ViV)

Most contemporary ViV procedures use balloon-expandable valves delivered transseptally, and they have shown high procedural success and acceptable survival in high-risk patients, representing a more established therapy than ViR. A recent multicenter registry study including 4243 patients reported a technical success rate of 97% and 30-day mortality of 4.3%. Procedural complications were infrequent: cardiac perforation 1.0%, conversion to open-heart surgery 0.7%, device embolization 0.18%, and device migration 0.3%. Overall, 99.2% of patients had none or mild MR at discharge, 30 days, and 1 year. The mean transmitral gradients after MViV were 6.7 mmHg at discharge and 7.4 mmHg at 1 year. One-year mortality was 13%^59^.

Although 1-year mitral valve reintervention rate was low (0–2.8%)^56,59,60^, this possibly only reflects the high-risk profile and inoperability of these patients, and durability beyond one year remains uncertain.

Despite its less invasive nature, transcatheter mitral ViV has several important limitations. In the Valve-in-Valve International Data (VIVID) registry, 61% of ViV patients had residual mean gradient ≥5 mmHg, and 8.2% had ≥10 mmHg despite successful implantation. Smaller true internal diameters of the surgical valve were strongly associated with higher residual gradients^61^.LVOT obstruction can be a significant complication after ViV. Contemporary studies report an LVOT obstruction incidence of 0–2.3% in carefully selected patients, which is significantly lower than the incidence observed after ViR^56,59,61,62^. Three principal mechanisms can cause LVOT obstruction during mitral ViV implantation. The first relates to cardiac morphology such as a small left ventricle, prominent septal hypertrophy, or a narrow aorto-mitral angle, which inherently predispose to a restricted outflow tract. The second involves factors intrinsic to the failed surgical bioprosthesis; valves that protrude toward the ventricular cavity, particularly bovine pericardial bioprostheses with a higher profile and leaflets that expand straight rather than folding outward as seen in porcine valves, are more likely to encroach upon the LVOT. The third mechanism is ventricular malposition of the transcatheter valve, in which implantation that is too deep causes the stent frame to project into the LVOT^63^. Therefore, meticulous preprocedural CT screening and precise positioning of the transcatheter valve are essential.

## Reintervention after TEER

Recurrent MR after TEER for PMR appears to result from a combination of progressive valve degeneration, device-related factors, and suboptimal procedural results. Progressive myxomatous degeneration may lead to worsening prolapse or flail at the clipped segment. Device- or procedure-related mechanisms include single-leaflet device attachment or leaflet injury at the clip site^64^. In addition, residual moderate MR at the end of the index procedure is a strong predictor of subsequent recurrence^65^. Although secondary MR is also included, U.S. Medicare data provide useful insight: among 11,396 patients who underwent TEER, 548 patients (4.8%) required a reintervention after a median of 4.5 months. Of these reinterventions, 294 patients (53.7%) underwent repeat TEER, whereas 254 patients (46.3%) underwent mitral valve surgery. The overall 30-day mortality was 8.6% (Redo TEER: 8.2%, mitral valve surgery: 9.1%)^66^.

### Redo TEER

Redo TEER can be considered in selected PMR anatomies, such as those with adequate leaflet width and length for secure grasping, preserved leaflet mobility, and acceptable transmitral gradients. Two contemporary series reported zero 30-day mortality, even with secondary MR included; however, in the Medicare series, the 30-day mortality was 8.2%^66^. This discrepancy likely reflects substantial differences in patient profiles. Reduction of MR to moderate or less was achieved in 79–100%^67,68^, although achieving mild or less MR was limited to 39% (also including secondary MR)^68^. At 1 year, event rates were lower in PMR than in SMR, suggesting a more favorable early clinical course in degenerative disease^68^. Although long-term durability remains uncertain, redo TEER is generally more feasible in PMR than in secondary MR.

### Surgery after TEER

Surgery after TEER in PMR remains technically demanding. Registry data from the STS database and the CUTTING-EDGE investigators consistently show that, although patients with PMR are more likely than those with secondary MR to undergo repair, surgical repair after failed TEER is achieved in only a small minority of cases (7–11% in the PMR subgroup), because leaflet perforation, tissue loss, scarring, and clip-related distortion often preclude a durable reconstruction^69,70^. However, when sufficient leaflet tissue remains, such as in anatomies with redundant or preserved leaflet segments, the clip can be removed and a durable repair becomes more feasible. Operative and early mortality are substantially higher than for de novo PMR repair: in national STS data the observed mortality for isolated mitral surgery after TEER is about 10%^69^, and in the multicenter CUTTING-EDGE registry 30-day and 1-year mortality rates are 17% and 31%, respectively^69^. Survival was better in PMR than in SMR, with 1-year mortality of 23% versus 38% and 3-year survival of 80% versus 59%^71^.

### Transcatheter mitral valve replacement (TMVR) after TEER

This complex procedure is an emerging option for patients who are unsuitable for redo surgery or repeat TEER. Early multicenter experience, primarily using a hybrid ELASTA-Clip laceration technique followed by transapical TMVR, reported a technical success rate of 95.4%^73^. In this hybrid approach, a dual-catheter electrosurgical system is positioned on the ventricular side of the clipped leaflet complex, and controlled current is applied to divide the tissue bridge along the anterior leaflet while preserving the attachment of all clips to the posterior leaflet, thereby creating an open central landing zone for the transcatheter valve^72^. At 30 days, 82% of patients had mild or less residual MR, but the 30-day rate of ischemic stroke was high at 15%^73^, and long-term durability remains uncertain. Because current evidence is limited to small, highly selected cohorts and TMVR after TEER is not an established reintervention pathway, it should be considered a rescue therapy for anatomically or surgically prohibitive cases rather than a factor that should influence the design of the index PMR repair. (Table 1).

**Table 1:** Summary of clinical outcomes for interventions and reinterventions in primary mitral regurgitation

Treatment modality	Key references	Patient cohort/setting	Main clinical outcomes and key constraints
Index: Surgical MV repair	David TE (2019) Brescia AA (2021)	Degenerative PMR (long-term follow-up)	*Outcomes*: 15-year probability of recurrent moderate/severe MR is 12.5%. 15-year reoperation-free survival is ~60%. Comparable clinical durability across anterior, posterior, and bileaflet prolapse in expert centers. *Constraints*: Efficacy highly dependent on surgical expertise and preoperative anatomy. Operative risk may be high in frail or elderly patients.
Index: Transcatheter Edge-to-Edge Repair (TEER)	Makkar RR (2023) Feldman T (2015)	Prohibitive/High-risk PMR with favorable grasping anatomy	*Outcomes*: 1-year MR success: 88.9%, 1-year mortality: 15.4% (STS/ACC TVT registry). 5-year effectiveness (EVEREST II) remains inferior to surgery (44% vs 64%). *Constraints*: Strict anatomical requirements (e.g., adequate grasping length, flail gap). Risk of iatrogenic mitral stenosis and single-leaflet device attachment.
Index: Transcatheter Mitral Valve Replacement (TMVR)	Zahr F (2023) Hell MM (2024)	High-risk PMR unsuitable for TEER	*Outcomes*: Excellent early safety in highly selected transfemoral cohorts (0% 30-day mortality). Real-world transapical data (Tendyne) show 9.3% 30-day and 28.6% 1-year mortality. *Constraints*: High risk of LVOT obstruction; off-label use significantly increases mortality. Routine use as an index intervention requires further validation.
Reintervention: Redo Surgery (Repair or MVR)	Smith RL (2025) Mehaffey JH (2018)	Failed index repair or degenerated MVR	*Outcomes*: Redo repair achieves high procedural success with very low early mortality (0–2%) in expert centers. Redo MVR provides definitive MR elimination but carries higher early mortality (2.6–15%) and lower long-term survival. *Constraints*: Re-entry injury risk, cumulative morbidity of multiple sternotomies, and prosthesis trade-offs (anticoagulation vs. degeneration).
Reintervention: Transcatheter Valve-in-Valve (ViV)	Goel K (2024) Simonato M (2021)	High-risk patients with degenerated bioprostheses	*Outcomes*: High technical success (97%), 30-day mortality: 4.3%, 1-year mortality: 13%. >99% achieve mild or less MR at 1 year. *Constraints*: Risk of elevated residual gradients (mean ~7 mmHg) and LVOT obstruction. Highly dependent on the true internal diameter and design of the index surgical valve.
Reintervention: Transcatheter Valve-in-Ring (ViR)	Guerrero M (2024) Simonato M (2021)	High-risk patients with failed surgical repair	*Outcomes*: Technical success ranges from 67–88% (lower than ViV). 30-day mortality: 7–8%, 1-year mortality: 22–29%. *Constraints*: High risk of underexpansion, paravalvular leak, device migration, and LVOT obstruction. Heavily dictated by the geometry and rigidity of the index annuloplasty ring.
Reintervention: Redo TEER	Sugiura A (2020) Kaneko T (2024)	Failed TEER in PMR patients with acceptable anatomy	*Outcomes*: 30-day mortality ranges from 0% (highly selected series) to 8.2% (Medicare data). MR reduction to moderate or less achieved in 79–100%. *Constraints*: Long-term durability is uncertain. Requires preserved leaflet mobility, adequate tissue width/length, and acceptable pre-procedural transmitral gradients.
Reintervention: Surgery after TEER	Chikwe J (2021) Kaneko T (2021)	Failed TEER requiring surgical rescue	*Outcomes*: Surgical repair achieved in only a small minority (7–11% in PMR). High 30-day mortality (10–17%) and 1-year mortality (23–31%). *Constraints*: Leaflet perforation, tissue loss, scarring, and clip-related distortion often preclude durable reconstruction, necessitating MVR.

This table consolidates key clinical data, including technical success, mortality, long-term durability, and primary constraints, for both surgical and transcatheter approaches across index and redo procedures.

## Designing the first intervention with future options in mind

**Fig. 1:**
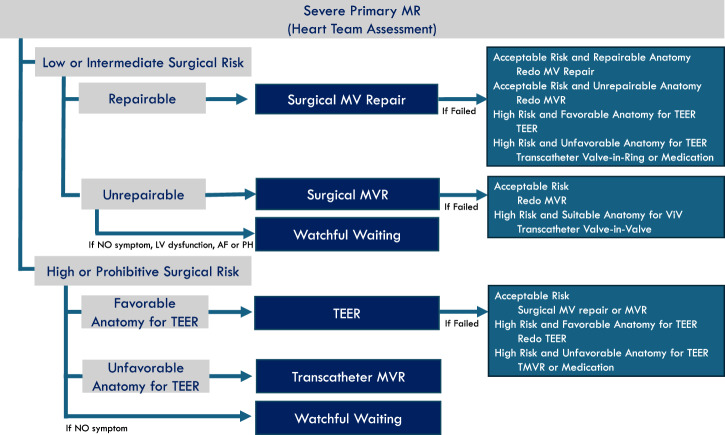
Interventional pathways in the lifetime management of severe primary mitral regurgitation. This algorithm outlines the decision-making process for index interventions based on operative risk and anatomical suitability, and delineates the subsequent transcatheter, surgical, and medical pathways following the failure or degeneration of the initial therapy.

Although achieving a durable result at the index intervention is the most important determinant of lifetime management in PMR, the repair strategy chosen at the first operation also profoundly influences the feasibility and safety of future reinterventions. A well-designed initial procedure can preserve multiple exit pathways—redo repair, replacement, TEER, valve-in-ring (ViR), or future transcatheter therapies—whereas certain technical decisions may unintentionally narrow or eliminate those options. (Fig. 1).

### Mitral valve repair: preserving future pathways

The technical design of surgical repair should avoid creating an anatomic configuration that limits reintervention.

- Annuloplasty ring selection. Undersized rings increase the risk of postoperative MS, complicate redo repair, make replacement more hazardous, and predispose TEER or ViR to high gradients; sizing to the anterior leaflet and inter-commissural distance mitigates these limitations. Ring geometry and rigidity should be tailored to the index repair and native valve physiology. Although rigid rings or incomplete flexible rings are generally unfavorable for ViR, ViR is not yet an established reintervention pathway; therefore, choosing a ring solely to “anticipate a potential future ViR” is not justified.
- Avoid excessive leaflet resection or too many edge-to-edge sutures. These maneuvers can reduce leaflet mobility and shorten coaptation surface, resulting in a “small valve” phenotype that is poorly suited to redo repair, difficult for TEER grasping, and highly vulnerable to stenosis after any type of reinterventions.
- Preserve leaflet height and tissue whenever feasible. A repair that maintains generous leaflet tissue and coaptation depth not only improves durability but also keeps options open for future chordal repair, or edge-to-edge repair.

### Mitral valve replacement: planning for future valve-in-valve

When repair is not feasible and MVR is selected, the choice of prosthesis and surgical technique should anticipate the likelihood of future valve-in-valve (ViV) procedures.

- Avoid prostheses that are too small for body size. Small true internal diameters markedly increase the risk of post-ViV MS.
- Implant the surgical valve in a left-atrial–oriented position, when anatomy permits, to minimize LVOT obstruction risk during future ViV.
- Direct the valve struts toward the apex rather than the septum, thereby maintaining a wide aorto–mitral angle, reducing anterior displacement, and lowering the predicted neo-LVOT gradient.

### TEER: considerations for future interventions

As with surgical repair, TEER requires careful avoidance of MS and the achievement of durable MR reduction; however, when future interventions must be preserved, additional considerations apply:

- Adequate leaflet tissue must be preserved for possible redo TEER or surgical re-repair. Excessive manipulation—such as repeatedly grasping the same segment or implanting more clips than necessary—can lead to leaflet scarring, reduced mobility, and restricted future options.
- Avoid creating high transmitral gradients. A clip strategy that leaves a small residual orifice compromises both surgery and redo TEER.

### Minimally invasive access in lifetime planning

Minimally invasive and robotic approaches should be viewed not only as tools to accelerate postoperative recovery but also as components of lifetime management. When the index operation is performed through a right mini-thoracotomy access, mediastinal adhesions behind the sternum are minimized, which can lower the hazard of a future re-entry and simplify subsequent median sternotomy if it becomes necessary years later. Equally, if the index operation was a sternotomy, a redo procedure via right mini-thoracotomy access can limit the dissection field, reduce adhesiolysis-related risk, and still allow high-quality repair or replacement in experienced hands^51,52^.

The foremost principle remains unchanged: do not compromise durability for access. Minimally invasive/robotic repair should achieve the same goals as sternotomy—durable elimination of MR with a generous coaptation zone, low transmitral gradient and no SAM. When performed by experienced surgeons in high-volume programs, minimally invasive/robotic approaches can offer the short-term benefits (faster recovery, earlier return to activity) and a plausible long-term advantage by preserving safer options for reintervention. In this sense, access planning is part of lifetime planning: choose the approach that maximizes repair durability today while keeping future surgical access as safe and flexible as possible.

### Long-term surveillance and holistic care across the lifespan

Beyond procedural decision-making, lifetime management of PMR also requires structured follow-up within dedicated heart valve clinics^1^. Patients with severe PMR managed conservatively require regular clinical and echocardiographic surveillance, whereas follow-up after intervention should be tailored to the procedure performed and to the presence of residual MR, prosthetic dysfunction, arrhythmias, or ventricular impairment. Management of atrial fibrillation and heart failure is integral to long-term care, as atrial fibrillation in PMR is associated with worse long-term outcomes and may compromise repair durability^74–76^. Control of broader cardiovascular risk factors, particularly blood pressure management and weight optimization, should also be integrated into longitudinal care. While direct evidence linking these factors to the prevention of recurrent primary MR is lacking, minimizing left ventricular afterload and preventing obesity-associated atrial or ventricular remodeling are mechanistically sound strategies to reduce hemodynamic stress on the index repair or prosthesis. Furthermore, in patients with prior valve repair, prosthetic material, or transcatheter devices, prevention of infective endocarditis remains crucial: contemporary guidance emphasizes meticulous oral hygiene, regular dental review, and antibiotic prophylaxis before at-risk dental procedures^77^.

## Conclusions

PMR is one of the few valve diseases in which a timely, durable repair can restore near-normal life expectancy. Lifetime management therefore, begins with early guideline-directed referral and a repair-first strategy before irreversible cardiac remodeling occurs. For patients whose anatomy or operative risk preclude durable reconstruction, alternative index options such as valve replacement or transcatheter edge-to-edge repair are available. When index interventions fail or degenerate, a broad surgical and transcatheter toolbox allows tailored reinterventions. However, the feasibility and safety of these future procedures are strongly dictated by the design of the initial operation. Crucially, true lifetime management extends beyond procedural execution, demanding lifelong clinical and echocardiographic surveillance, vigilant arrhythmia management, and stringent cardiovascular risk factor control. Overall, PMR care must shift from a single interventional event to a holistic, longitudinal Heart Team strategy that balances immediate durability, preserved future options, and comprehensive medical care.
